# Two-Dimensional DOA Estimation for Incoherently Distributed Sources with Uniform Rectangular Arrays

**DOI:** 10.3390/s18113600

**Published:** 2018-10-23

**Authors:** Tao Wu, Zhenghong Deng, Yiwen Li, Yijie Huang

**Affiliations:** 1 School of Automation, Northwestern Polytechnical University, Xi’an 710072, China; hyjrly@126.com; 2 Science and Technology on Combustion, Thermal-Structure and Internal Flow Laboratory, Northwestern Polytechnical University, Xi’an 710072, China; lee_yiwen@nwpu.edu.cn

**Keywords:** incoherently distributed sources, generalized steering matrix, generalized signal vector, rotational invariance relations, angle matching

## Abstract

Aiming at the two-dimensional (2D) incoherently distributed (ID) sources, we explore a direction-of-arrival (DOA) estimation algorithm based on uniform rectangular arrays (URA). By means of Taylor series expansion of steering vector, rotational invariance relations with regard to nominal azimuth and nominal elevation between subarrays are deduced under the assumption of small angular spreads and small sensors distance firstly; then received signal vectors can be described by generalized steering matrices and generalized signal vectors; thus, an estimation of signal parameters via rotational invariance techniques (ESPRIT) like algorithm is proposed to estimate nominal elevation and nominal azimuth respectively using covariance matrices of constructed subarrays. Angle matching method is proposed by virtue of Capon principle lastly. The proposed method can estimate multiple 2D ID sources without spectral searching and without information of angular power distribution function of sources. Investigating different *SNR,* sources with different angular power density functions, sources in boundary region, distance between sensors and number of sources, simulations are conducted to investigate the effectiveness of the proposed method.

## 1. Introduction

In most applications of array signal processing, traditional DOA estimation is based on point source models which can simplify calculations. Point source models assume that propagations between sources and receive arrays are straight paths and the spatial characteristics of sources can be ignored. In the field of wireless communication, there are obstacles around sources; the propagations from sources to receive arrays are multipath. In the real surrounding of underwater, on the one hand, there exist paths from seabed and sea surface of backscatters; on the other hand, spatial scatterers of targets cannot be ignored when the distances from targets and receive arrays are short. Considering the spatial scatterers and multipath phenomena, point models cannot characterize sources effectively, which should be described by distributed source models [1]. The signal of a source only propagates from a single direction through a straight path under the assumption of point source model. Distributed sources can be regarded as an assembly of point sources within a spatial distribution. The shape of spatial distribution is related to geometry and surface property of a target in underwater detection for instance. Spatial distribution of a distributed source can be generally modeled as Gaussian or uniform with parameters containing nominal angles and angular spreads. Nominal angles represent the center of targets and angular spreads represent the spatial extension of targets.

According to the scatterers coherence of sources, distributed sources can be classified into coherently distributed (CD) sources and incoherently distributed (ID) sources [1]. CD sources suppose that scatterers within a source are coherent whereas scatterers of an ID source are assumed to be uncorrelated. In this paper, ID sources are considered.

For CD sources, representative achievements of DOA estimation are methods extended from point source models through rotation invariance relations with respect to different array configuration under the small angular spreads assumption [2,3,4,5,6,7,8,9,10]. As to ID sources, people have presented subspace-based algorithms such as distributed signal parameter estimator (DSPE) [1] and dispersed signal parametric estimation (DSPARE) [11] which are developed from multiple signal classification (MUSIC). Based on uniform linear arrays (ULA), the authors of [12] have extended Capon method for ID sources, where two-dimensional (2D) spectral searches based on high-order matrix inversion are involved. The authors of [13] have proposed a generalized Capon’s method by introducing a matrix pencil of sample covariance matrix and normalized covariance matrix to traditional Capon framework. A robust generalized Capon’s method in [14] has been proposed by supplementing a constraint function using property of covariance matrix to cost function of algorithm in [12], which has better accuracy without a priori knowledge of shape of ID sources. Maximum likelihood (ML) approaches [15,16,17] have better accuracy but require high computational complexity. The authors of [18] have taken the lead in extending covariance matching estimation techniques (COMET) to DOA estimation of ID sources using ULA. They have converted complex nonlinear optimization to two successive one-dimensional (1D) searches utilizing the extended invariance principle (EXIP). Considering Gaussian and uniform ID sources, number of sensors in experiment of [18] ranges from 4 to 20; they have proved that when sensors is increased to a certain extent with other parameters fixed, estimation tends to be less accurate. Applying Taylor series expansion of steering vector to approximate the array covariance matrix using the central moments of the source, the authors of [19] have elaborated a lower computational COMET for ID sources, which exhibits a good performance at low *SNR* based on ULA with 11 sensors. The authors of [20] have presented the ambiguity problem of COMET-EXIP algorithm using ULA and proposed an inequality constraint in the original objective functions to solve this problem. The authors of [21] have turned covariance matching problem to retrieve parameters of received covariance by exploiting the annihilating property of linear nested arrays. The authors of [22] have embedded algorithm of [19] into a Kalman filter for DOA tracking problem of ID sources.

The aforementioned DOA estimations of ID sources consider the sources and receive arrays are in the same plane where sources are described by 1D model with two parameters: nominal angle and angular spread. Generally, sources are in different plane with receive arrays, which should be modeled as two-dimensional (2D) models with parameters as nominal azimuth angle, nominal elevation angle, azimuth spread, and elevation spread. With more parameters, there have been relatively few studies on estimation of 2D ID sources. The authors of [23] have extended COMET algorithm to 2D scenarios, which separates variables of each source based on alternating projection technique, then formulate equations set of unknown variables. The algorithm from [23] is applicable to any arrays in three-dimensional spaces but requires considerably high computational complexity. In addition, experiments of [23] have only considered a relatively small number of targets. The authors of [24] have proposed a two-stage approach based on rotation invariance relations of generalized steering vector of double parallel uniform linear arrays (DPULA), where nominal elevation is firstly estimated via TLS-ESPRIT, then nominal azimuth is acquired by 1D searching.

In order to estimate DOA of 2D ID sources, we propose an algorithm based on URA. Through Taylor series expansions of steering vectors, received signal vectors of arrays can be expressed as a generalized form which is combination of generalized steering matrix and generalized signal vector under the assumption of small distance of sensors and small angular spread. Consequently, the rotation invariance relations of constructed subarrays with respect to nominal azimuth and nominal elevation are derived. Constructed subarrays fully use elements of URA, so the estimation accuracy is improved. Then the nominal azimuth and nominal elevation can be calculated respectively by means of an ESPRIT like algorithm. Thus, estimation of multiple 2D ID sources do not need spectral searching and avoid high computational complexity. Lastly, the angle matching method is proposed according to Capon spectral search. Without information of angular power distribution function, the proposed method can detect sources with different angular power distribution functions.

## 2. Arrays Configuration and Signal Model

**Figure 1:**
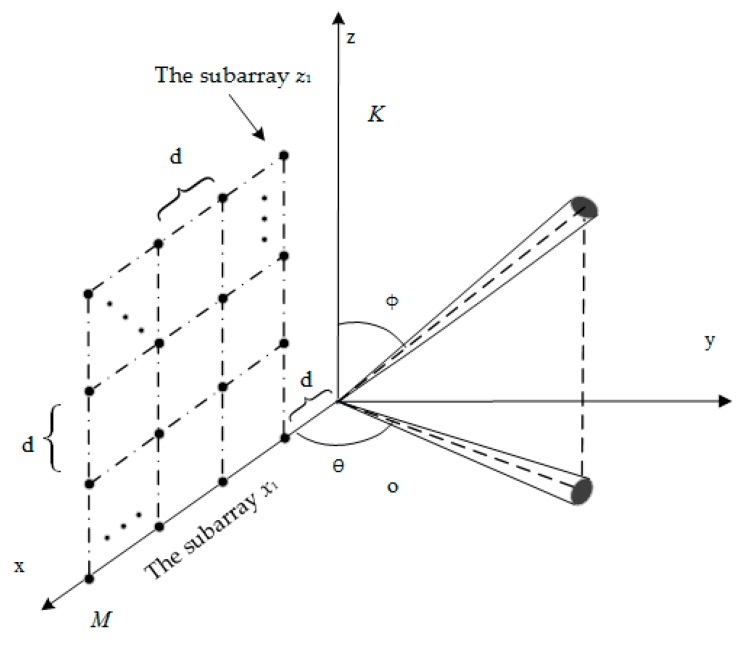
Uniform rectangular arrays configuration.

As shown in Figure 1, the URA consists of *M* × *K* sensors on *xoz* plane. The distance between sensors along the direction of *x* and *z* axes is set at *d* meters. Azimuth *θ* and elevation *φ* are used to describe the direction of signal in a three dimensional space. There are *q* 2D ID narrowband sources with nominal angles (*θ_i_*, *φ_i_*) (*i* = 1, 2, …, *q*) denoting nominal azimuth and nominal elevation of the *i*th sources impinging into arrays. *θ_i_* ∈[0,π], *φ_i_* ∈[0,π]. *λ* is the wavelength of the impinging signal. The additive noise is considered as Gaussian white with zero mean and uncorrelated with sensors.

The *M ×* 1 dimensional received vector of the *M* sensors located on the ***x***-axis defined as subarray ***x***_1_ can be written as

(1) $$x_{1}(t)=\sum_{i=1}^{q}\iint \alpha_{1}(\theta ,\varphi )s_{i}(\theta ,\varphi ,t)d\theta d\varphi +n_{x1}(t),$$

where **n***_x_*_1_(*t*) denotes the noise vector of subarray. The *M* × 1 dimensional vector **α**_1_(*θ*,*φ*) denotes the steering vector of subarray ***x***_1_ with respect to point source, which can be expressed as

(2) $$\alpha_{1}(\theta ,\varphi )={\left[e^{j2\pi d\cos\theta \sin\varphi /\lambda },e^{j2\pi 2d\cos\theta \sin\varphi /\lambda },\cdots ,e^{j2\pi Md\cos\theta \sin\varphi /\lambda }\right]}^{T},$$

*s_i_*(*θ*,*φ*,*t*) is the complex random angular signal density of the distributed source representing the reflection intensity of the source from angle (*θ*,*φ*) at the snapshot index *t*. Unlike a point source, signal of a distributed source exists not only in the direction of (*θ_i_*,*φ_i_*) but also in a spatial distribution around (*θ_i_*,*φ_i_*). A distributed source is defined as incoherently distributed if *s_i_*(*θ*,*φ*,*t*) from one direction is uncorrelated with other directions, which can be modeled as a random process as

(3) $$E\;[s_{i}(\theta ,\varphi ,t)s_{i}{}^{*}(\theta {}^{\prime },\varphi {}^{\prime },t)]=P_{i}f_{i}(\theta ,\varphi ;u)\delta (\theta -\theta {}^{\prime })\delta (\varphi -\varphi {}^{\prime }),$$

where *δ*(·) is the Kronecker delta function, *P_i_* is the power of the source, *f_i_*(*θ*,*φ*;**u***_i_*) is its normalized angular power density function (APDF) reflecting geometry and surface property of a distributed source. APDF is determined by parameter set **u***_i_*. For a Gaussian ID source, **u***_i_* = [$\theta_{i}$, $\phi_{i}$, $\sigma_{\theta i}$, $\;\sigma_{\phi i}$, ρ] denoting nominal azimuth, nominal elevation, azimuth spread, elevation spread, and covariance coefficient respectively, APDF can be expressed as

(4) $$f_{i}(\theta ,\varphi ;u_{i})=\frac{1}{2\pi \sigma_{\theta i}\sigma_{\varphi i}\sqrt{1-\rho^{2}}}\exp\left\{-\frac{1}{2\sigma_{\theta i}\sigma_{\varphi i}(1-\rho^{2})}\left[{\left(\frac{\theta -\theta_{i}}{\sigma_{\theta i}}\right)}^{2}-2\rho \frac{\left(\theta -\theta_{i})(\varphi -\varphi_{i}\right)}{\sigma_{\theta i}\sigma_{\varphi i}}+{\left(\frac{\varphi -\varphi_{i}}{\sigma_{\varphi i}}\right)}^{2}\right]\right\}$$

For a uniform ID source, **u***_i_* = [$\theta_{i}$, $\phi_{i}$, $\sigma_{\theta i}$, $\sigma_{\phi i}$] denoting nominal azimuth, nominal elevation, azimuth spread and elevation spread respectively. APDF can be expressed as

(5) $$f_{i}(\theta ,\varphi ;u_{i})=\left\{ \begin{array}{ll} \frac{1}{4\sigma_{\theta i}\sigma_{\varphi i}} & \left|\theta -\theta_{i}\right|\leq \sigma_{\theta i}\;and\;\left|\varphi -\varphi_{i}\right|\leq \sigma_{\varphi i}\\ 0 & \left|\theta -\theta_{i}\right|\geq \sigma_{\theta i}\;or\;\left|\varphi -\varphi_{i}\right|\geq \sigma_{\varphi i} \end{array}\right..$$

The *M ×* 1 dimensional received vector of the *k*th subarray parallel to ***x***-axis, which is defined as subarray ***x****_k_,* can be written as

(6) $$x_{k}(t)=\sum_{i=1}^{q}\iint \alpha_{k}(\theta ,\varphi )s_{i}(\theta ,\varphi ,t)d\theta d\varphi +n_{xk}(t),$$

where **α***_k_*(*θ*,*φ*) denotes the steering vector of subarray ***x****_k_* with respect to point source, which can be written as

(7) $$\alpha_{k}(\theta ,\varphi )=\alpha_{1}(\theta ,\varphi )e^{j2\pi (k-1)d\cos\varphi /\lambda }.$$

The *M* × *K* dimensional received matrix of the URA along the ***x***-axis can be expressed as

(8) $$X(t)={\left[x_{1}(t),x_{2}(t),\cdots ,x_{K}(t)\right]}^{T}.$$

The *K ×* 1 dimensional received vector of the *K* sensors with distance *d* and parallel to ***z***-axis defined as subarray ***z***_1_ can be written as

(9) $$z_{1}(t)=\sum_{i=1}^{q}\iint \beta_{1}(\theta ,\varphi )s_{i}(\theta ,\varphi ,t)d\theta d\varphi +n_{z1}(t),$$

where the *K* × 1 dimensional vector **β**_1_(*θ,φ*) denotes the steering vector of subarray ***z***_1_ with respect to point source, **n***_z_*_1_(*t*) denotes the noise vector of subarray ***z***_1_. **β**_1_(*θ,φ*) can be expressed as

(10) $$\beta_{1}(\theta ,\varphi )=e^{j2\pi d\cos\theta \sin\varphi /\lambda }{\left[1,e^{j2\pi d\cos\varphi /\lambda },\cdots ,e^{j2\pi (K-1)d\cos\varphi /\lambda }\right]}^{T}.$$

The *K* × 1 dimensional received vector of the *m*th subarray parallel to ***z***-axis defined as subarray ***z****_m_* can be written as

(11) $$z_{m}(t)=\sum_{i=1}^{q}\iint \beta_{m}(\theta ,\varphi )s_{i}(\theta ,\varphi ,t)d\theta d\varphi +n_{zm}(t),$$

where **β***_m_*(*θ*,*φ*) denotes the steering vector of subarray ***z****_m_* with respect to point source, which can be written as

(12) $$\beta_{m}(\theta ,\varphi )=\beta_{1}(\theta ,\varphi )e^{j2\pi (m-1)d\cos\theta \sin\varphi /\lambda }.$$

The *K* × *M* dimensional received matrix of the URA along the ***z***-axis can be expressed as

(13) $$Z(t)={\left[z_{1}(t),z_{2}(t),\cdots ,z_{M}(t)\right]}^{T}.$$

## 3. Proposed Method

This section consists of five parts. First of all, generalized steering matrices are obtained from taking the first-order Taylor series expansions of steering vectors under the assumption of small angular spreads and small distance *d* of the URA. Then, the rotation invariance relations of generalized steering matrices within subarrays are derived. In the next part, the received signal vectors are transformed as combinations of generalized signal vectors and generalized steering matrices. Next, based on rotation invariance relations of constructed subarrays, nominal azimuth and nominal elevation can be estimated separately by an ESPRIT like algorithm. Afterwards, angle matching method is proposed based on the Capon principle. Lastly computational procedure is summarized and complexity analysis is analyzed with comparison of two existing methods for 2D ID sources.

### 3.1. Generalized Steering Matrix

Assume that angular spread of distributed sources is small, take the first-order Taylor series expansions of steering vectors of subarrays ***x****_k_* and ***x****_k−_*_1_ at (*θ_i_*, *φ_i_*)*,* we have

(14) $$\alpha_{k-1}(\theta ,\varphi )\approx \alpha_{k-1}(\theta_{i},\varphi_{i})+[\alpha_{k-1}{(\theta_{i},\varphi_{i}){]}^{\prime }}_{\theta }(\theta -\theta_{i})+[\alpha_{k-1}{(\theta_{i},\varphi_{i}){]}^{\prime }}_{\varphi }(\varphi -\varphi_{i}),$$

(15) $$\alpha_{k}(\theta ,\varphi )\approx \alpha_{k}(\theta_{i},\varphi_{i})+[\alpha_{k}{(\theta_{i},\varphi_{i}){]}^{\prime }}_{\theta }(\theta -\theta_{i})+[\alpha_{k}{(\theta_{i},\varphi_{i}){]}^{\prime }}_{\varphi }(\varphi -\varphi_{i}),$$

where ${[\cdot {]}^{\prime }}_{\theta }$ and ${[\cdot {]}^{\prime }}_{\varphi }$ denote the first-order partial derivatives of *θ* and *φ* at (*θ_i_*, *φ_i_*), the following relationship can be obtained from Equation (7)

(16) $$\alpha_{k}(\theta_{i},\varphi_{i})=\alpha_{k-1}(\theta_{i},\varphi_{i})e^{j2\pi d\cos\varphi_{i}/\lambda },$$

(17) $$[\alpha_{k}{(\theta_{i},\varphi_{i}){]}^{\prime }}_{\theta }={[\alpha_{k-1}(\theta_{i},\varphi_{i}){]}^{\prime }}_{\theta }e^{j2\pi d\cos\varphi_{i}/\lambda },$$

(18) $$[\alpha_{k}{(\theta_{i},\varphi_{i}){]}^{\prime }}_{\varphi }={[\alpha_{k-1}(\theta_{i},\varphi_{i}){]}^{\prime }}_{\varphi }e^{j2\pi d\cos\varphi_{i}/\lambda }+(-j2\pi d\sin\varphi_{i}/\lambda )e^{j2\pi (k-1)d\cos\varphi_{i}/\lambda }\alpha_{k-1}(\theta_{i},\varphi_{i}).$$

If *d/λ* ≪ 0.5, the second term of the right side of the Equation (18) can be ignored, so Equation (18) can be written as

(19) $$[\alpha_{k}{(\theta_{i},\varphi_{i}){]}^{\prime }}_{\varphi }\approx {[\alpha_{k-1}(\theta_{i},\varphi_{i}){]}^{\prime }}_{\varphi }e^{j2\pi d\cos\varphi_{i}/\lambda }.$$

In the point source model case, steering vectors is used to describe response of an array. Nevertheless, as for distribute sources, generalized steering vectors or generalized steering matrices [5,24] represent response of arrays. Define *M* × 3*q* dimensional generalized steering matrix of subarray ***x***_1_ as $\left[A_{11},A_{12},A_{13}\right]$. **A**_11_, **A**_12_, and **A**_13_ are expressed as

(20) $$\left\{ \begin{array}{l} A_{11}=[\alpha_{1}(\theta_{1},\varphi_{1}),\alpha_{1}(\theta_{2},\varphi_{2}),\cdots ,\alpha_{1}(\theta_{q},\varphi_{q})]\\ A_{12}=\left[{[\alpha_{1}(\theta_{1},\varphi_{1}){]}^{\prime }}_{\theta },{[\alpha_{1}(\theta_{2},\varphi_{2}){]}^{\prime }}_{\theta },\cdots ,{[\alpha_{1}(\theta_{q},\varphi_{q}){]}^{\prime }}_{\theta }\right]\\ A_{13}=\left[{[\alpha_{1}(\theta_{1},\varphi_{1}){]}^{\prime }}_{\varphi },{[\alpha_{1}(\theta_{2},\varphi_{2}){]}^{\prime }}_{\varphi },\cdots ,{[\alpha_{1}(\theta_{q},\varphi_{q}){]}^{\prime }}_{\varphi }\right] \end{array}\right..$$

Define the generalized steering matrix of subarray ***x****_k_* as $\left[A_{k1},A_{k2},A_{k3}\right]$,

(21) $$\left\{ \begin{array}{l} A_{k1}=[\alpha_{k}(\theta_{1},\varphi_{1}),\alpha_{k}(\theta_{2},\varphi_{2}),\cdots ,\alpha_{k}(\theta_{q},\varphi_{q})]\\ A_{k2}=\left[{[\alpha_{k}(\theta_{1},\varphi_{1}){]}^{\prime }}_{\theta },{[\alpha_{k}(\theta_{2},\varphi_{2}){]}^{\prime }}_{\theta },\cdots ,{[\alpha_{k}(\theta_{q},\varphi_{q}){]}^{\prime }}_{\theta }\right]\\ A_{k3}=\left[{[\alpha_{k}(\theta_{1},\varphi_{1}){]}^{\prime }}_{\varphi },{[\alpha_{k}(\theta_{2},\varphi_{2}){]}^{\prime }}_{\varphi },\cdots ,{[\alpha_{k}(\theta_{q},\varphi_{q}){]}^{\prime }}_{\varphi }\right] \end{array}\right..$$

From Equations (16), (17) and (19) we can obtain the generalized steering matrix of ***x****_k_* subarray as

(22) $$[A_{k1},A_{k2},A_{k3}]\approx [A_{11}\Phi_{z}^{k-1},A_{12}\Phi_{z}^{k-1},A_{13}\Phi_{z}^{k-1}],$$

where **Φ***_z_* is rotation invariance operator, which can be written as

(23) $$\Phi_{z}=diag(e^{j2\pi d\cos\varphi_{1}/\lambda },e^{j2\pi d\cos\varphi_{2}/\lambda },\cdots ,e^{j2\pi d\cos\varphi_{q}/\lambda }).$$

Take the first-order Taylor series expansions of steering vectors of subarrays ***z****_m_* and ***z****_m−_*_1_ at (*θ_i_*, *φ_i_*), we have

(24) $$\beta_{m-1}(\theta ,\varphi )\approx \beta_{m-1}(\theta_{i},\varphi_{i})+[\beta_{m-1}{(\theta_{i},\varphi_{i}){]}^{\prime }}_{\theta }(\theta -\theta_{i})+[\beta_{m-1}{(\theta_{i},\varphi_{i}){]}^{\prime }}_{\varphi }(\varphi -\varphi_{i}),$$

(25) $$\beta_{m}(\theta ,\varphi )\approx \beta_{m}(\theta_{i},\varphi_{i})+[\beta_{m}{(\theta_{i},\varphi_{i}){]}^{\prime }}_{\theta }(\theta -\theta_{i})+[\beta_{m}{(\theta_{i},\varphi_{i}){]}^{\prime }}_{\varphi }(\varphi -\varphi ).$$

Form Equation (12) we have

(26) $$\beta_{m}(\theta ,\varphi )=\beta_{m-1}(\theta_{i},\varphi_{i})e^{j2\pi d\cos\theta_{i}\sin\varphi_{i}/\lambda },$$

(27) $${[\beta_{m}(\theta_{i},\varphi_{i}){]}^{\prime }}_{\theta }={[\beta_{m-1}(\theta_{i},\varphi_{i}){]}^{\prime }}_{\theta }e^{j2\pi d\cos\theta_{i}\sin\varphi_{i}/\lambda }+(-j2\pi d\sin\theta_{i}\sin\varphi_{i}/\lambda )e^{j2\pi d\cos\theta_{i}\sin\varphi_{i}/\lambda }\beta_{m-1}(\theta_{i},\varphi_{i}),$$

(28) $${[\beta_{m}(\theta_{i},\varphi_{i}){]}^{\prime }}_{\varphi }={[\beta_{m-1}(\theta_{i},\varphi_{i}){]}^{\prime }}_{\varphi }e^{j2\pi d\cos\theta_{i}\sin\varphi_{i}/\lambda }+(j2\pi d\cos\theta_{i}\cos\varphi_{i}/\lambda )e^{j2\pi d\cos\theta_{i}\sin\varphi_{i}/\lambda }\beta_{m-1}(\theta_{i},\varphi_{i}).$$

If d/λ ≪ 0.5, the second term of the right side of the Equations (27) and (28) can be ignored, so Equations (27) and (28) can be written as

(29) $${[\beta_{m}(\theta_{i},\varphi_{i}){]}^{\prime }}_{\theta }\approx {[\beta_{m-1}(\theta_{i},\varphi_{i}){]}^{\prime }}_{\theta }e^{j2\pi d\cos\theta_{i}\sin\varphi_{i}/\lambda },$$

(30) $${[\beta_{m}(\theta_{i},\varphi_{i}){]}^{\prime }}_{\varphi }\approx {[\beta_{m-1}(\theta_{i},\varphi_{i}){]}^{\prime }}_{\varphi }e^{j2\pi d\cos\theta_{i}\sin\varphi_{i}/\lambda }.$$

Define the *M* × 3*q* dimensional generalized steering matrix of subarray ***z***_1_ as $\left[B_{11},B_{12},B_{13}\right]$; **B**_11_, **B**_12_, and **B**_13_ are written as

(31) $$\left\{ \begin{array}{l} B_{11}=[\beta_{1}(\theta_{1},\varphi_{1}),\beta_{1}(\theta_{2},\varphi_{2}),\cdots ,\beta_{1}(\theta_{q},\varphi_{q})]\\ B_{12}=\left[{[\beta_{1}(\theta_{1},\varphi_{1}){]}^{\prime }}_{\theta },{[\beta_{1}(\theta_{2},\varphi_{2}){]}^{\prime }}_{\theta },\cdots ,{[\beta_{1}(\theta_{q},\varphi_{q}){]}^{\prime }}_{\theta }\right]\\ B_{13}=\left[{[\beta_{1}(\theta_{1},\varphi_{1}){]}^{\prime }}_{\varphi },{[\beta_{1}(\theta_{2},\varphi_{2}){]}^{\prime }}_{\varphi },\cdots ,{[\beta_{1}(\theta_{q},\varphi_{q}){]}^{\prime }}_{\varphi }\right] \end{array}\right..$$

Define the generalized steering matrix of subarray ***z***_1_ as $\left[B_{m1},B_{m2},B_{m3}\right]$,

(32) $$\left\{ \begin{array}{l} B_{m1}=[\beta_{m}(\theta_{1},\varphi_{1}),\beta_{m}(\theta_{2},\varphi_{2}),\cdots ,\beta_{m}(\theta_{q},\varphi_{q})]\\ B_{m2}=\left[{[\beta_{m}(\theta_{1},\varphi_{1}){]}^{\prime }}_{\theta },{[\beta_{m}(\theta_{2},\varphi_{2}){]}^{\prime }}_{\theta },\cdots ,{[\beta_{m}(\theta_{q},\varphi_{q}){]}^{\prime }}_{\theta }\right]\\ B_{m3}=\left[{[\beta_{m}(\theta_{1},\varphi_{1}){]}^{\prime }}_{\varphi },{[\beta_{m}(\theta_{2},\varphi_{2}){]}^{\prime }}_{\varphi },\cdots ,{[\beta_{m}(\theta_{q},\varphi_{q}){]}^{\prime }}_{\varphi }\right] \end{array}\right..$$

From Equations (26), (29), and (30), the generalized steering matrix of ***z****_m_* $\left[B_{m1},B_{m2},B_{m3}\right]$ can be expressed as

(33) $$[B_{m1},B_{m2},B_{m3}]\approx [B_{11}\Phi_{x}^{m-1},B_{12}\Phi_{x}^{m-1},B_{13}\Phi_{x}^{m-1}],$$

where **Φ***_x_* is rotation invariance operator, which can be written as

(34) $$\Phi_{x}=diag(e^{j2\pi d\cos\theta_{1}\sin\varphi_{1}/\lambda },e^{j2\pi d\cos\theta_{2}\sin\varphi_{2}/\lambda },\cdots ,e^{j2\pi d\cos\theta_{q}\sin\varphi_{q}/\lambda }).$$

### 3.2. Generalized Signal Vector

Define generalized signal vector as $\bar{s}=[{\bar{s}}_{1},{\bar{s}}_{2},{\bar{s}}_{3}]$*^H^*; ${\bar{s}}_{1},{\bar{s}}_{2}$ and ${\bar{s}}_{3}$ are written as

(35) $$\left\{ \begin{array}{l} {\bar{s}}_{1}=[\rho_{10},\rho_{20},\cdots ,\rho_{k0}]\\ {\bar{s}}_{2}=[\rho_{1\theta },\rho_{2\theta },\cdots ,\rho_{k\theta }]\\ {\bar{s}}_{3}=[\rho_{1\varphi },\rho_{2\varphi },\cdots ,\rho_{k\varphi }] \end{array}\right.,$$

where

(36) $$\left\{ \begin{array}{l} \rho_{i0}=\iint s_{i}(\theta ,\varphi ,t)d\theta d\varphi \\ \rho_{i\theta }=\iint (\theta -\theta_{i})s_{i}(\theta ,\varphi ,t)d\theta d\varphi \\ \rho_{i\varphi }=\iint (\varphi -\varphi_{i})s_{i}(\theta ,\varphi ,t)d\theta d\varphi \end{array}\right..$$

Substitute Taylor series expansions (15) into Equation (6), we obtain

(37) $$\begin{array}{ll} x_{k}(t) & \approx \sum_{i=1}^{q}\iint \alpha_{k}(\theta_{i},\varphi_{i})s_{i}(\theta ,\varphi ,t)d\theta d\varphi +\sum_{i=1}^{q}\iint [\alpha_{k}{(\theta_{i},\varphi_{i}){]}^{\prime }}_{\theta }(\theta -\theta_{i})s_{i}(\theta ,\varphi ,t)d\theta d\varphi \\ & +\sum_{i=1}^{q}\iint [\alpha_{k}{(\theta_{i},\varphi_{i}){]}^{\prime }}_{\varphi }(\varphi -\varphi_{i})s_{i}(\theta ,\varphi ,t)d\theta d\varphi +n_{xk}(t)\\ & =\sum_{i=1}^{q}\alpha_{k}(\theta_{i},\varphi_{i})\rho_{i0}+\sum_{i=1}^{q}[\alpha_{k}(\theta_{i},\varphi_{i}){]}^{\prime }\rho_{i\theta }+\sum_{i=1}^{q}[\alpha_{k}{(\theta_{i},\varphi_{i}){]}^{\prime }}_{\varphi }\rho_{i\varphi }+n_{xk}(t) \end{array}$$

Thus, received vector of subarray ***x****_k_* can be expressed as a combination of the generalized signal vector and generalized steering matrix

(38) $$x_{k}(t)\approx [A_{k1},A_{k2},A_{k3}]\bar{s}+n_{xk}(t).$$

The *MK* × 1 received vector of the URA along the ***x***-axis can be express as

(39) $$\bar{X}(t)={\left[x_{1}{\left(t\right)}^{T},x_{2}{\left(t\right)}^{T},\cdots ,x_{K}{\left(t\right)}^{T}\right]}^{T}\approx \left[\begin{array}{ccc} A_{11}, & A_{12}, & A_{13}\\ A_{11}\Phi_{z}, & A_{12}\Phi_{z}, & A_{13}\Phi_{z}\\ & \cdots & \\ A_{11}\Phi_{z}^{K-1}, & A_{12}\Phi_{z}^{K-1}, & A_{13}\Phi_{z}^{K-1} \end{array}\right]\bar{s}+n_{X}(t),$$

where **n***_X_*(*t*) is the noise vector of the URA along the ***x***-axis, which can be expressed as

(40) $$n_{X}(t)={\left[n_{x1}{\left(t\right)}^{T},n_{x2}{\left(t\right)}^{T},\cdots ,n_{xK}{\left(t\right)}^{T}\right]}^{T}.$$

Similarly, the received vector of subarray ***z****_m_* can be expressed as

(41) $$z_{m}(t)\approx [B_{m1},B_{m2},B_{m3}]\bar{s}+n_{z1}(t).$$

Supposing different ID sources are uncorrelated there exist following relations within generalized signal vectors (see Appendix A)

(42) $$E[\rho_{il}\rho_{in}^{*}]=\left\{ \begin{array}{ll} P_{i} & l=n=0\\ P_{i}M_{\theta i} & l=n=\theta \\ P_{i}M_{\varphi i} & l=n=\varphi \\ 0 & l\neq n \end{array}\right.,$$

where *M_θi_*, *M_φi_* can be express as

(43) $$M_{\theta i}=\iint {\left(\theta -\theta_{i}\right)}^{2}p_{i}(\theta ,\varphi ,t)d\theta d\varphi ,$$

(44) $$M_{\varphi i}=\iint {\left(\varphi -\varphi_{i}\right)}^{2}p_{i}(\theta ,\varphi ,t)d\theta d\varphi .$$

According to the assumption that different ID sources are uncorrelated, so we have

(45) $$E\;[\bar{s}{\bar{s}}^{H}]=diag(\Lambda ,M_{\theta }\Lambda ,M_{\varphi }\Lambda ),$$

where **Λ**, **M***_θ_*, and **M***_φ_* can be written as

(46) $$\left\{ \begin{array}{l} \Lambda =diag(\sigma_{s1}^{2},\sigma_{s2}^{2},\cdots ,\sigma_{sq}^{2})\\ M_{\theta }=diag(M_{\theta 1},M_{\theta 2},\cdots ,M_{\theta q})\\ M_{\varphi }=diag(M_{\varphi 1},M_{\varphi 2},\cdots ,M_{\varphi q}) \end{array}\right..$$

### 3.3. Nominal Angles Estimation

We construct two subarrays **X**_1_ and **X**_2_ along the direction of the ***x***-axis. **X**_1_ is constituted by arrays from ***x***_1_ to ***x****_K−_*_1_; while **X**_2_ contains arrays from ***x***_2_ to ***x****_K_*. Thus, **X**_1_(*t*) is *M*(*K −* 1) × 1 dimensional received vector of **X**_1_ also equals a vector containing elements from 1 to *MK − M* row of $\bar{X}$(*t*); whereas **X**_2_(*t*) is *M*(*K −* 1) × 1 dimensional received vector of **X**_2_ containing elements from *M +* 1 to *MK* of $\bar{X}$(*t*). **X**_1_(*t*) and **X**_2_(*t*) can be written as

(47) $$\left\{ \begin{array}{l} X_{1}(t)={\left[x_{1}{\left(t\right)}^{T},x_{2}{\left(t\right)}^{T},\cdots ,x_{K-1}{\left(t\right)}^{T}\right]}^{T}=\left[A_{x1}^{1},A_{x2}^{1},A_{x3}^{1}\right]\bar{s}+n_{X1}(t)\\ X_{2}(t)={\left[x_{2}{\left(t\right)}^{T},x_{2}{\left(t\right)}^{T},\cdots ,x_{K}{\left(t\right)}^{T}\right]}^{T}=\left[A_{x1}^{2},A_{x2}^{2},A_{x3}^{2}\right]\bar{s}+n_{X2}(t) \end{array}\right.,$$

**n***_X_*_1_(*t*) is noise vector of **X**_1_ containing elements from 1 to *MK − M* row of **n***_X_*(*t*), **n***_X_*_2_(*t*) is noise vector of **X**_2_ containing elements from *M* to *MK* row of **n***_X_*(*t*). $\left[A_{x1}^{1},A_{x2}^{1},A_{x3}^{1}\right]$ is the generalized steering matrix of subarray **X**_1_(*t*), which can be expressed as

(48) $$\left[A_{x1}^{1},A_{x2}^{1},A_{x3}^{1}\right]=\left[\begin{array}{ccc} A_{11}, & A_{12}, & A_{13}\\ A_{11}\Phi_{z}, & A_{12}\Phi_{z}, & A_{13}\Phi_{z}\\ & \cdots & \\ A_{11}\Phi_{z}^{K-2}, & A_{12}\Phi_{z}^{K-2}, & A_{13}\Phi_{z}^{K-2} \end{array}\right],$$

$\left[A_{x1}^{2},A_{x2}^{2},A_{x3}^{2}\right]$ is the generalized steering matrix of subarray **X**_2_(*t*). From Equation (22) we can obtain

(49) $$\left[A_{x1}^{2},A_{x2}^{2},A_{x3}^{2}\right]\approx \left[A_{x1}^{1}\Phi_{z},A_{x2}^{1}\Phi_{z},A_{x3}^{1}\Phi_{z}\right],$$

which proves the rotational invariance relation between the two constructed subarrays **X**_1_ and **X**_2_. According to the ESPRIT principle, combing the vector **X**_1_(*t*) and **X**_2_(*t*), we obtain a vector with rotational invariance property as

(50) $$X_{12}(t)=\left[\begin{array}{l} X_{1}(t)\\ X_{2}(t) \end{array}\right]=\left[A_{x1},A_{x2},A_{x3}\right]\bar{s}+n_{X12}(t),$$

where the generalized steering matrix of the combination **X**_12_(*t*) can be expressed as

(51) $$\left[A_{x1},A_{x2},A_{x3}\right]=\left[\begin{array}{l} A_{x1}^{1},A_{x2}^{1},A_{x3}^{1}\\ A_{x1}^{2},A_{x2}^{2},A_{x3}^{2} \end{array}\right].$$

The combination of noise vector can be expressed as

(52) $$n_{X12}(t)=\left[\begin{array}{l} n_{X1}(t)\\ n_{X2}(t) \end{array}\right].$$

ESPRIT framework is based on rotational invariance property of signal subspace, which can be derived from the rotational invariance property of the received signal vector. Signal subspace can be acquired by eigendecomposition of the covariance matrix of received vector **X**_12_(*t*) which can be expressed as

(53) $$R_{x}^{12}=E\;[X_{12}(t)X_{12}^{H}(t)].$$

$R_{x}^{12}$ can be replaced by sample covariance matrix with *N* snapshots as

(54) $${\hat{R}}_{x}^{12}=\frac{1}{N}\sum_{t=1}^{N}X_{12}(t)X_{12}^{H}(t).$$

From Equations (45) and (46) we find that eigenvalues of covariance matrix of received signal vector consist of three parts **Λ,** **M***_θ_***Λ** and **M***_φ_***Λ**. The three parts all has *q* elements. Each part corresponds to respective eigenvectors. Under the assumption of small angular spread, we can obtain *M_θi_* < 1 and *M_φi_* < 1. Therefore, subspace spanned by eigenvectors corresponding to the largest *q* eigenvalues is equal to subspace spanned by **A***_x_*_1_. Suppose **E***_x_* is 2*M*(*K* − 1) × *q* dimensional matrix with columns as the eigenvectors of the covariance matrix $R_{x}^{12}$ corresponding to the *q* largest eigenvalues. Accordingly, there exists a *q* × *q* nonsingular matrix **T** satisfying the following relation

(55) $$E_{x}=\left[\begin{array}{l} A_{x1}^{1}\\ A_{x1}^{1}\Phi_{z} \end{array}\right]T.$$

Let **E***_x_*_1_ and **E***_x_*_2_ denote matrices selecting upper and lower *MK − M* rows of **E***_x_*

(56) $$\left\{ \begin{array}{l} E_{x1}=A_{x1}^{1}T\\ E_{x2}=A_{x1}^{2}T \end{array}\right..$$

So we have

(57) $$E_{x2}=E_{x1}T{\;}^{-1}\Phi_{z}T.$$

Let

(58) $$\Omega_{x}=T{\;}^{-1}\Phi_{z}T.$$

Thus, the eigenvalues of **Φ***_x_* is elements of **Ф***_z_*. **Ω***_x_* can be obtained as

(59) $$\Omega_{x}=E_{x1}^{+}E_{x2},$$

where (•)^+^ denotes pseudo-inverse operator, then the nominal elevation of the sources can be get from

(60) $$\varphi_{i}=\arccos\frac{angle(\eta_{i})}{2\pi d/\lambda }i=1,2,\cdots ,q,$$

where *η_i_* is the *i*th eigenvalues of **Ω***_x_*, *angle*(•) denotes argument of complex variable. 

Similarly, we construct two subarrays **Z**_1_ and **Z**_2_ along the direction of ***z***-axis. **Z**_1_ is constituted by arrays from ***z***_1_ to ***z****_M−_*_1_; while **Z**_2_ contains arrays from ***z***_2_ to ***z****_M_*. Z_1_(*t*) is *K*(*M −* 1) × 1 dimensional receive vector of **Z**_1_ containing elements from 1 to *K*(*M* − 1) row of $\bar{Z}$(*t*); whereas **Z**_2_(*t*) is *K*(*M −* 1) × 1 dimensional received vector of **Z**_2_ containing elements from *K +* 1 to *MK* row of $\bar{Z}$(*t*). **n***_Z_*_1_(*t*) and **n***_Z_*_2_(*t*) have the similar definition as **n***_X_*_1_(*t*) and **n***_X_*_2_(*t*). The received vectors **Z**_1_(*t*) and **Z**_2_(*t*) can be expressed as

(61) $$\left\{ \begin{array}{l} Z_{1}(t)={\left[z_{1}{\left(t\right)}^{T},z_{2}{\left(t\right)}^{T},\cdots ,z_{M-1}{\left(t\right)}^{T}\right]}^{T}=\left[B_{z1}^{1},B_{z2}^{1},B_{z3}^{1}\right]\bar{s}+n_{Z1}(t)\\ Z_{2}(t)={\left[z_{2}{\left(t\right)}^{T},z_{2}{\left(t\right)}^{T},\cdots ,z_{M}{\left(t\right)}^{T}\right]}^{T}=\left[B_{z1}^{2},B_{z2}^{2},B_{z3}^{2}\right]\bar{s}+n_{Z2}(t) \end{array}\right.,$$

where $\left[B_{z1}^{1},B_{z2}^{1},B_{z3}^{1}\right]$ is the generalized steering matrix of **Z**_1_(*t*), which can be expressed as

(62) $$\left[B_{z1}^{1},B_{z2}^{1},B_{z3}^{1}\right]=\left[\begin{array}{ccc} B_{11}, & B_{12}, & B_{13}\\ B_{11}\Phi_{x}, & B_{12}\Phi_{x}, & B_{13}\Phi_{x}\\ & \cdots & \\ B_{11}\Phi_{x}^{M-2}, & B_{12}\Phi_{x}^{M-2}, & B_{13}\Phi_{x}^{M-2} \end{array}\right].$$

Form Equation (33), the generalized steering matrix of **Z**_2_(*t*) $\left[B_{z1}^{2},B_{z2}^{2},B_{z3}^{2}\right]$ can be obtained as

(63) $$\left[B_{z1}^{2},B_{z2}^{2},B_{z3}^{2}\right]\approx \left[B_{z1}^{1}\Phi_{x},B_{z2}^{1}\Phi_{x},B_{z3}^{1}\Phi_{x}\right],$$

which proves the rotational invariance relation between the two constructed subarrays **Z**_1_ and **Z**_2_. Combing the vector **Z**_1_(*t*) and **Z**_2_(*t*), we obtain a vector with rotational invariance property as

(64) $$Z_{12}(t)=\left[\begin{array}{l} Z_{1}(t)\\ Z_{2}(t) \end{array}\right]=\left[{Β}_{z1},{Β}_{z2},{Β}_{z3}\right]\bar{s}+n_{Z12}(t),$$

where the generalized steering matrix of **Z**_12_(*t*) can be expressed as

(65) $$\left[{Β}_{z1},{Β}_{z2},{Β}_{z3}\right]=\left[\begin{array}{l} B_{z1}^{1},B_{z2}^{1},B_{z3}^{1}\\ B_{z1}^{2},B_{z2}^{2},B_{z3}^{2} \end{array}\right].$$

The combination of noise vector of **Z**_1_(*t*) and **Z**_2_(*t*) can be expressed as

(66) $$n_{Z12}(t)=\left[\begin{array}{l} n_{Z1}(t)\\ n_{Z2}(t) \end{array}\right].$$

The covariance matrices of received signal **Z**_12_(*t*) can be expressed as

(67) $$R_{z}^{12}=E[Z_{12}(t)Z_{12}^{H}(t)].$$

which can be replaced by sample covariance matrix with *N* snapshots as

(68) $${\hat{R}}_{z}^{12}=\frac{1}{N}\sum_{t=1}^{N}Z_{12}(t)Z_{12}^{H}(t).$$

Suppose **E***_z_* is 2*K*(*M −* 1) × *q* matrix with columns as the eigenvectors of the covariance matrix $R_{z}^{12}$ corresponding to the *q* largest eigenvalues. As the same as **E***_x_*, **E***_z_* is the same subspace spanned by $B_{z1}$. Accordingly, there exists a *q* × *q* nonsingular matrix **Q** satisfying the following relation

(69) $$E_{z}=\left[\begin{array}{l} B_{11}\\ B_{11}\Phi_{x} \end{array}\right]Q.$$

Let **E***_z_*_1_ and **E***_z_*_2_ denote matrices selecting upper and lower *MK − K* rows of **E***_z_*

(70) $$\left\{ \begin{array}{l} E_{z1}=B_{z1}^{1}Q\\ E_{z2}=B_{z1}^{1}Q \end{array}\right..$$

So we have

(71) $$E_{z2}=E_{z1}Q^{-1}\Phi_{x}Q.$$

Let

(72) $$\Omega_{z}=Q^{-1}\Phi_{x}Q.$$

Thus, the eigenvalues of **Φ***_z_* is elements of **Ф***_x_*. **Ω***_z_* can be obtained as

(73) $$\Omega_{z}=E_{z1}^{+}E_{z2}.$$

Then the nominal azimuth of the sources can be get from

(74) $$\theta_{i}=\arccos\frac{angle(\mu_{i})}{2\pi d/\lambda \sin\varphi_{i}}i=1,2,\cdots ,q,$$

where *μ_i_* is the *i*th eigenvalues of **Ω***_z_*.

### 3.4. Angle Matching Method

After all the eigenvalues of **Ω***_x_* and **Ω***_z_* are calculated, we need to match the right *θ_i_* to right *φ_i_*. We can obtain cost function by applying Capon principle to subarray ***x***_1_ firstly. Then angle matching can be obtained by substituting all the possible pairs into the cost function. 

The generalized steering matrix of subarray ***x***_1_ is $\left[A_{11},A_{12},A_{13}\right]$, the Capon principle with regard to the subarray ***x***_1_ can be expressed as

(75) $$\min w^{H}R_{x}^{1}w\;subject\;to\;w^{H}\left[A_{11},A_{12},A_{13}\right]=1.$$

where $R_{x}^{1}$ is the covariance matrix of subarray ***x***_1_. Equation (75) can be solved through minimization of Lagrange function as follows

(76) $$L(\theta ,\varphi )=w^{H}R_{x}^{1}w\;+\lambda \left\{w^{H}\left[A_{11},A_{12},A_{13}\right]-1\right\},$$

Take the derivative of the Equation (76) with regard to **w** and set the result equal to 0, we obtain

(77) $$R_{x}^{1}w\;=-\left[A_{11},A_{12},A_{13}\right]\lambda ,$$

Considering constraint condition, we obtain

(78) $$\lambda =-{\left\{{\left[A_{11},A_{12},A_{13}\right]}^{H}{\left(R_{x}^{1}\right)}^{-1}\left[A_{11},A_{12},A_{13}\right]\right\}}^{-1},$$

The optimal vector **w***_opt_* can be obtained as follows

(79) $$w_{opt}=-\frac{{\left(R_{x}^{1}\right)}^{-1}\left[A_{11},A_{12},A_{13}\right]}{{\left[A_{11},A_{12},A_{13}\right]}^{H}{\left(R_{x}^{1}\right)}^{-1}\left[A_{11},A_{12},A_{13}\right]},$$

Then, the cost function can be expressed as

(80) $$V(\theta ,\varphi )=\frac{1}{{\left[A_{11},A_{12},A_{13}\right]}^{H}{\left(R_{x}^{1}\right)}^{-1}\left[A_{11},A_{12},A_{13}\right]}.$$

$R_{x}^{1}$ can be replaced by sample covariance matrix with *N* snapshots as

(81) $${\hat{R}}_{x}^{1}=\frac{1}{N}\sum_{t=1}^{N}x_{1}(t)x_{1}^{H}(t).$$

Suppose that all eigenvalues of **Ω**_x_ and **Ω**_z_ are calculated. Now we summarize the angle matching procedure as follows:

Step 1: Calculate nominal elevation *φ_i_* (*i* = 1, 2,…, *q*) from Equation (60). Select one elevation angle ${\hat{\varphi }}_{i}=\varphi_{i}$ form set $\left\{\varphi_{1},\varphi_{2},\cdots ,\varphi_{q}\right\}$ at random. Superscript *_^_* denotes the angle already determined. Substitute ${\hat{\varphi }}_{i}$ to the eigenvalues set of **Ω**_z_:$\left\{angle(\mu_{1}),angle(\mu_{2}),\cdots ,angle(\mu_{q})\right\}$ and calculate *q* matching azimuth angles $\theta_{j}$ (j=1,2,⋯,q) from Equation (74). So we get *q* possible pairs $\left(\theta_{j},{\hat{\varphi }}_{i}\right)$ (j=1,2,⋯,q).

Step 2: As the right parameters of sources can meet the objective function in Equation (75). Substitute $\left(\theta_{j},{\hat{\varphi }}_{i}\right)$ (j=1,2,⋯,q) into cost function (80). Choose the azimuth $\theta_{j}$ making $V(\theta_{j},{\hat{\varphi }}_{i})$ reach the maximum as the right azimuth angle matching to ${\hat{\varphi }}_{i}$, which is labeled as ${\hat{\theta }}_{i}$.

Step 3: Delete ${\hat{\varphi }}_{i}$ from set $\left\{\varphi_{1},\varphi_{2},\cdots ,\varphi_{q}\right\}$ meanwhile delete eigenvalue $e^{j2\pi d/\lambda \cos{\hat{\theta }}_{i}\sin{\hat{\varphi }}_{i}}$ from set $\{angle(\mu_{1}),angle(\mu_{2}),\cdots ,$ $angle(\mu_{q})\}$.

Step 4: Set *q* = *q* − 1.

Step 5: Repeat steps 1 to 4.

All the nominal azimuth and nominal elevation can be matched after (*q* + 2)(*q* − 1)/2 times calculation.

### 3.5. Computational Procedure and Complexity Analysis

Now, our algorithm can be summarized as follows

Step 1: Compute sample covariance matrices ${\hat{R}}_{x}^{12}$ and ${\hat{R}}_{z}^{12}$ using Equations (54) and (68).

Step 2: Find the eigenvectors **E***_x_* and **E***_z_* corresponding to the *q* largest eigenvalues through eigendecomposition of ${\hat{R}}_{x}^{12}$ and ${\hat{R}}_{z}^{12}$. Divide **E***_x_* into **E***_x_*_1_, **E***_x_*_2_ and divide **E***_z_* into **E***_z_*_1_, **E***_z_*_2_.

Step 3: Obtain **Ω**_x_ and **Ω**_z_ from Equations (59) and (73), calculate eigenvalues $\mu_{i}$ and $\eta_{i}$ (*i* = 1, 2, …, *q*) through eigendecomposition of **Ω***_x_* and **Ω***_z_*.

Step 4: Calculate nominal azimuth *θ_i_* and nominal elevation *φ_i_* from Equations (60) and (74).

Step 5: Compute sample covariance matrix ${\hat{R}}_{x}^{1}$ form Equation (81) and take the angle matching procedure.

We analyze the computational complexity of the proposed method in comparison with COMET [23] which can be applied for URA and Zhou’s algorithm [24] which uses DPULA for 2D ID sources. COMET [23] is a method under alternating projection algorithm framework, its computational cost mostly consists of the calculation of the sample covariance matrix which needs *O*(*NM*^2^*K*^2^) and the alternating projection technique with respect to cost functions which is *O*(*M*^4^*K*^4^ + *2M*^2^*K*^2^). Zhou’s algorithm uses TLS-ESPRIT to calculate nominal elevation and 1D searching to find nominal azimuth. Computational cost of Zhou’s algorithm [24] mainly contains calculation of the sample covariance matrix *O*(4*NM*^2^), eigendecomposition and inversion of the sample covariance matrix *O*(16*M*^3^) and 1D searching *O*(8*M*^3^). Computational cost of the proposed algorithm mainly contains calculation of the sample covariance matrix *O*[*N*(*MK* − *K*)^2^ + *N*(*MK* − *M*)^2^], eigendecomposition of ${\hat{R}}_{x}^{12}$ and ${\hat{R}}_{z}^{12}$ *O*[8 (*MK* − *K*)^3^ + 8(*MK − M*)^3^], eigendecomposition of **Ω**_x_ and **Ω**_z_ *O*(*q^3^*) and angle matching *O*(*q^2^*). The main computational complexity of the three algorithms are shown in Table 1.

From Table 1, we can conclude that the computational cost of the proposed algorithm is higher than Zhou’s algorithm [24] when *K* > 2 and lower than COMET algorithm [23] when the two algorithm use same number of sensors.

## 4. Results and Discussion

In this section, the effectiveness of the proposed algorithm is investigated through five simulation experiments. The array configuration is shown in Figure 1. Root mean squared error (*RMSE*) is applied for the evaluation of the performance of estimation. *RMSE_θ_* and *RMSE_φ_* denote the *RMSE* of nominal azimuth and nominal elevation respectively, which can be expressed as

(82) $$RMSE_{\theta }=\sqrt{\frac{1}{Mc}\sum_{\varsigma }^{Mc}{\left({\hat{\theta }}^{\varsigma }-\theta \right)}^{2}},$$

(83) $$RMSE_{\varphi }=\sqrt{\frac{1}{Mc}\sum_{\varsigma }^{Mc}{\left({\hat{\varphi }}^{\varsigma }-\varphi \right)}^{2}},$$

where *MC* is the number of Monte Carlo simulations. ${\hat{\theta }}_{i}^{\varsigma }$ and ${\hat{\varphi }}_{i}^{\varsigma }$ are the estimated nominal azimuth and nominal elevation of *i*th source in ςth Monte Carlo simulation.

**Figure 2:**
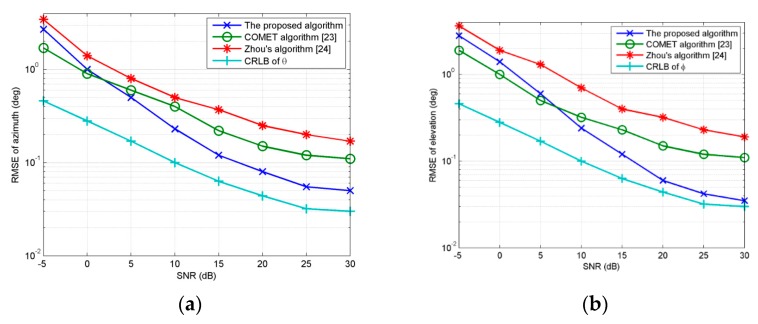
(**a**) *RMSE_θ_* estimated by three algorithms for 2D ID sources vesus *SNR*; (**b**) *RMSE_φ_* estimated by three algorithms for 2D ID sources vesus *SNR*.

In the first experiment, we examine the performance of the proposed method vesus *SNR* with regard to two Gaussian ID source with parameters [30°, 45°, 2°, 2°, 0.5] and [50°, 45°, 2°, 2°, 0.5]. The sources are supposed to have the same power. Snapshots number is set at 200, *MC* = 100, *M = K* = 8. *d* = *λ/*10. Figure 2a,b show *RMSE_θ_* and *RMSE_φ_* curves with *SNR* varying from −5 dB to 30 dB. The figures also show the estimation of the COMET [23] using the URA, Zhou’s algorithm [24] using arrays ***x***_1_ and ***x***_2_ and the Cramer–Rao lower bound (CRLB). As shown from Figure 2a,b, when *SNR* changes from −5 dB to 0 dB, COMET [23] presents better performance than the proposed algorithm. As *SNR* increases, *RMSE_θ_* and *RMSE_φ_* of all algorithms decrease. The proposed algorithm has better performance than other algorithms with *SNR* ranging from 10 dB to 30 dB. Thus, it can be concluded that our method has a good performance when *SNR* is at high levels. Estimation of our method is based on acquiring signal subspace through eigendecomposition of covariance matrix of received vector. The accuracy of eigendecomposition would deteriorate at low *SNR*, while COMET [23] separates the noise and signal power through covariance matching fitting matrix by alternating projection technique firstly, as a result, perform better at low *SNR.*

**Figure 3:**
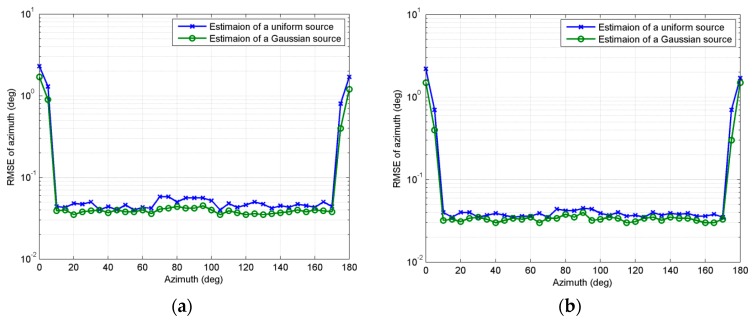
(**a**) *RMSE_θ_* estimated with *θ* changing from 0° to 180° while *φ* is fixed at 20°; (**b**) *RMSE_φ_* estimated with *φ* changing from 0° to 180° while *θ* is fixed at 20°.

In the second experiment, we investigate the estimation of source near the boundary region. Consider a Gaussian and a uniform ID source respectively. Both sources have angular spread $\sigma_{\theta }=\sigma_{\phi }=$2°. Covariance coefficients of Gaussian sources are set at 0.5. *SNR* is set at 15 dB and snapshots number is 200, *MC* = 100, *M = K* = 8, *d* = *λ/*10. Figure 3a shows *RMSE_θ_* curves as nominal azimuth changing from 0° to 180° with nominal elevation fixed at 20°, while Figure 3b shows *RMSE_φ_* curves as nominal elevation changing from 0° to 180° with nominal azimuth fixed at 20°. As can be seen, both *RMSE_θ_* and *RMSE_φ_* increase markedly near the boundary region. Generally, the error of the boundary region estimated by our method is acceptable.

**Figure 4:**
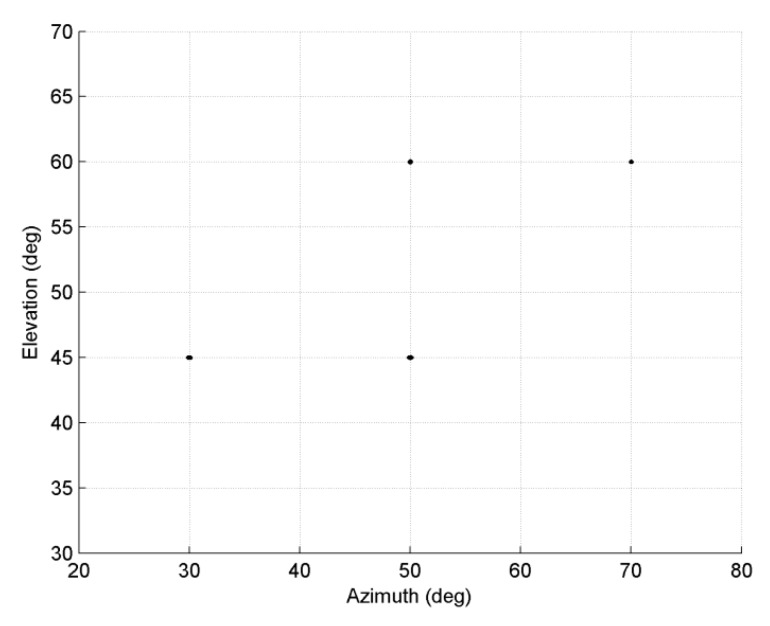
Estimated results for 2D ID sources with different angular power density functions.

In the third experiment, we examine the performance of the proposed method with regard to estimation of sources with different APDFs simultaneously. Consider four ID sources with same power and parameters of sources are set as [30°, 45°, 2°, 2°, 0.5], [50°, 45°, 1.5°, 3°, 0.5], [50°, 60°, 2°, 2.5°], and [70°, 60°, 1.5°, 3.5°]. The first and second sources are Gaussian whereas the third and fourth sources are uniform. The experiment set *SNR* at 15 dB; number of snapshots is 200. *MC =* 100, *M = K* = 8, *d* = *λ/*10. Figure 4 shows the estimated result of four sources, which indicate that the proposed method can estimate sources with different APDFs effectively.

**Figure 5:**
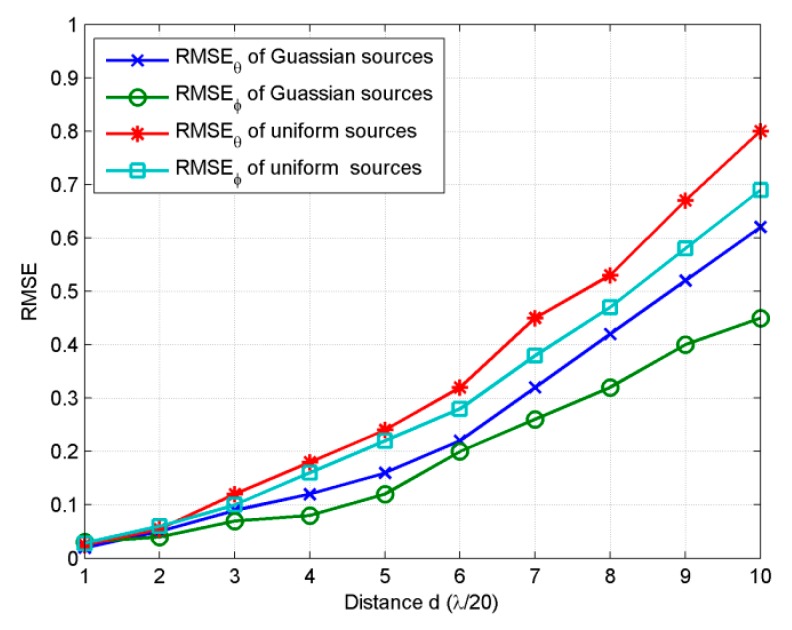
*RMSE* estimated for 2D ID sources with different distance *d*.

In the fourth experiment, we examine the performance of the proposed method vesus the distance *d* between adjacent sensors. As the rotational invariance relations of generalize steering matrices are obtained under the small distance *d* assumption, it is necessary to investigate the influence of *d* to the estimation. Four sources with the same parameters as the sources in third example are set to be estimated as the *d* varying from *λ/*20 to *λ/*2. *SNR* is set at 15 dB and snapshots number is 200, *MC* = 100, *M = K* = 8. Figure 5 shows that *RMSE_θ_* and *RMSE_φ_* of both Gaussian and uniform sources increase with the distance *d* from *λ/*20 to *λ/*2. When *d* is *λ/*2, *RMSE_θ_* and *RMSE_φ_* of Gaussian sources reach 0.62 and 0.44, those of uniform sources reach 0.79 and 0.67, which are still satisfactory results. It can be concluded that the proposed algorithm shows satisfactory performance with small distance between adjacent sensors.

According to the result of third example, the smaller distance *d* is, the higher estimation accuracy is. However, in the same channel the frequency increases, wavelength decreases. High frequency detection needs smaller *d* than low frequency detection to attain the same estimation accuracy. Actually, when the distance *d* is small, installation accuracy of sensors will get worse, which would also deteriorate the estimation accuracy. Thus, installation accuracy and the distance *d* is a matter of balance especially in high frequency detection. In the field of low frequency underwater detection, frequency of sonar can be down to 100 HZ, which means wavelength can reach 14.5 m approximately, on this condition *d*/λ can reach a small value practically.

In the fifth experiment, we investigate the performance of the proposed algorithm versus the number of sources and sensors. Theoretically, if *M = K*, the proposed algorithm can estimate *M*(*M* − 1) different ID sources and COMET [23] can estimate *M*^2^. Utilizing double parallel arrays which contains only 2*M* sensors can estimate *M* sources simultaneously. We consider seven URA with *M* varying from 4 to 10. The total number of sources is set at *M*^2^ which is a theoretical upper limit of the trail. The (*a*, *b*)th source is set at [20° + (*a −* 1)10°, 20° + (*b* − 1)10°]. All sources are Gaussian and have same power. *SNR* is 15 dB, number of snapshots is 200, *MC* = 100, *d* = λ/10, $\sigma_{\theta }=\sigma_{\phi }=$2°. Covariance coefficients of Gaussian sources are set at 0.5. When all sources are estimated successfully, the difference between estimators is accuracy which can be described by *RMSE*. When not all sources are estimated successfully, *RMSE* cannot differentiate performance of estimators. Thus, an indicator reflecting the number of sources detected is needed to measure the performance of different estimators. Estimation is regarded as effective when the estimated angles satisfying $\sqrt{{\left({\hat{\theta }}_{i}-\theta_{i}\right)}^{2}+{\left({\hat{\varphi }}_{i}-\varphi_{i}\right)}^{2}}\leq$ 5°. Define detection probability as *N*_d_/*M*^2^ where *N*_d_ is number of source estimated effectively. So in theory the detection probability of the proposed algorithm is (*M* − 1)/*M*, COMET is 1, Zhou’s algorithm [24] is 1/*M.*

**Figure 6:**
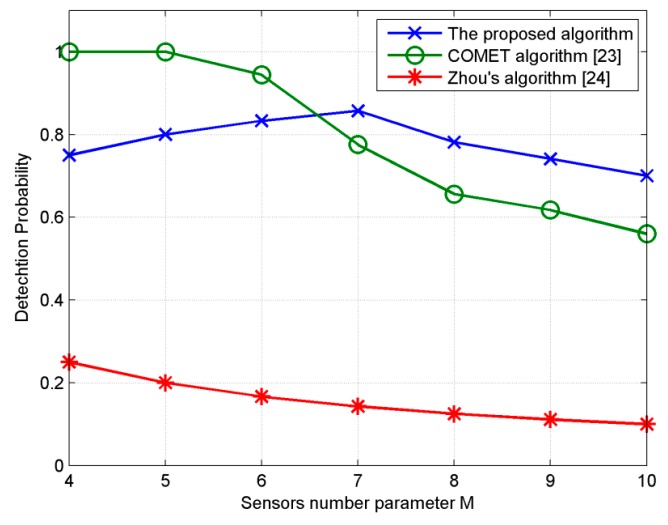
Detection probability by three algorithms with different numbers of sources and sensors.

As can be seen from Figure 6, the estimation probability of zhou’s algorithm [24] is consistent with theoretical value within all range of the trail, so are the estimation probabilities of our method and COMET with *M* < 6. When *M* ≥ 7, our method perform better than COMET, which means using 7 × 7 or larger arrays our method can estimated more sources than COMET if there are large number of sources that need estimating Involving eigendecomposition of high dimensional matrices, effectiveness of our method deteriorates as the number of sources becomes large. For instance, if *M* = 8 and total source is 64, the covariance matrix of received vector $R_{z}^{12}$ is 112 × 112 dimensional, **Ω***_x_* and **Ω***_z_* is 64 × 64 dimensional, which all need eigendecomposition. The deterioration of COMET [23] is also closely related to the number of sources. COMET [23] separates unknown variables of each source based on alternating projection technique, and then formulate equation sets of unknown variables. In the separating process, 64 inversion operations of a 64 × 64 dimensional matrix are executed on the condition *M* = 8 and total source is 64. Consequently, the errors of the separating process deliver to equations set and affect the validity of estimation.

## 5. Conclusions

In this paper, we propose an estimator for 2D ID sources using URA. The received signal vectors can be transformed as combinations of generalized signal vectors and generalized steering matrices through Taylor series expansions. By virtue of the rotational invariance relations of generalized steering matrices within subarrays, an ESPRIT like algorithm are introduced in detail, then angle matching method based on Capon principle is proposed. Simulations demonstrate the effectiveness of the proposed method with regard to sources with different APDF, distance *d* between sensors, sources in boundary region. Simulation also indicates that our method has better performance when *SNR* is at high level on the condition that the quantity of sources is relatively small and can estimate more sources in sufficient sources circumstance using large dimensional arrays.

## Figures and Tables

**Table 1:** Computational complexity of different methods

Method	Total
COMET algorithm [23]	*O*(*NM*^2^*K*^2^) + *O*(*M*^4^*K*^4^ + *2M*^2^*K*^2^)
Zhou’s algorithm [24]	*O*(4*NM*^2^) + *O*(16*M*^3^) + *O*(8*M*^3^)
Proposed algorithm	*O*[*N*(*MK* − *K*)^2^ + *N*(*MK* − *M*)^2^] + *O*[8(*MK − K*)^3^ + 8(*MK − M*)^3^] + *O*(*q^3^*) + *O*(*q^2^*)

## Appendix A

Take Gaussian distribution described by (4) for example, if *l = n* = 0, $E\;[\rho_{il}\rho_{in}^{*}]$ can be expressed as

(A1) $$\begin{array}{ll} E[\rho_{i0}\rho_{i0}^{*}] & =\iint E\;[s_{i}(\theta ,\varphi ,t)s_{i}^{*}(\theta ,\varphi ,t)]d\theta d\varphi \\ & =P_{i}\iint f_{i}(\theta ,\varphi ;u)d\theta d\varphi \\ & =P_{i} \end{array}.$$

If *l = n = θ*, $E[\rho_{il}\rho_{in}^{*}]$ can be expressed as

(A2) $$\begin{array}{ll} E[\rho_{i\theta }\rho_{i\theta }^{*}] & =P_{i}\iint E[(\theta -\theta_{i})s_{i}(\theta ,\varphi ,t)(\theta -\theta_{i})s_{i}^{*}(\theta ,\varphi ,t)]d\theta d\varphi \\ & =P_{i}\iint {\left(\theta -\theta_{i}\right)}^{2}f_{i}(\theta ,\varphi ,t)d\theta d\varphi \\ & =P_{i}M_{\theta i} \end{array},$$

where

(A3) $$\begin{array}{ll} M_{\theta i} & =\iint \frac{{\left(\theta -\theta_{i}\right)}^{2}}{2\pi \sigma_{\theta i}\sigma_{\varphi i}\sqrt{1-\rho^{2}}}\exp\left\{-\frac{1}{2\sigma_{\theta i}\sigma_{\varphi i}(1-\rho^{2})}\left[{\left(\frac{\theta -\theta_{i}}{\sigma_{\theta i}}\right)}^{2}-2\rho \frac{\left(\theta -\theta_{i})(\varphi -\varphi_{i}\right)}{\sigma_{\theta i}\sigma_{\varphi i}}+{\left(\frac{\varphi -\varphi_{i}}{\sigma_{\varphi i}}\right)}^{2}\right]\right\}d\theta d\varphi \\ & =\sigma_{\theta i}^{2} \end{array}.$$

Similarly, $E[\rho_{i\varphi }\rho_{i\varphi }]=P_{i}M_{\varphi i}$ and $M_{\varphi i}=\sigma_{\varphi i}^{2}$.

If *l ≠ n*, there exists six cases, *l* = 0 with *n = θ* or *φ*, *l* = *θ* with *n =* 0 or *φ* and *l* = *φ* with *n =* 0 or *θ.* Take *l* = *θ* with *n = φ* and *l* = *0* with *n = φ* for example, $E\;[\rho_{il}\rho_{in}^{*}]$ can be expressed as

(A4) $$\begin{array}{l} E[\rho_{i\theta }\rho_{i\varphi }^{*}]=P_{i}\iint E[(\theta -\theta_{i})s_{i}(\theta ,\varphi ,t)(\varphi -\varphi_{i})s_{i}^{*}(\theta ,\varphi ,t)]d\theta d\varphi \\ =P_{i}\iint (\theta -\theta_{i})(\varphi -\varphi_{i})f_{i}(\theta ,\varphi ,t)d\theta d\varphi \\ =\iint \frac{\left(\theta -\theta_{i})(\varphi -\varphi_{i}\right)}{2\pi \sigma_{\theta i}\sigma_{\varphi i}\sqrt{1-\rho^{2}}}\exp\left\{-\frac{1}{2\sigma_{\theta i}\sigma_{\varphi i}(1-\rho^{2})}\left[{\left(\frac{\theta -\theta_{i}}{\sigma_{\theta i}}\right)}^{2}-2\rho \frac{\left(\theta -\theta_{i})(\varphi -\varphi_{i}\right)}{\sigma_{\theta i}\sigma_{\varphi i}}+{\left(\frac{\varphi -\varphi_{i}}{\sigma_{\varphi i}}\right)}^{2}\right]\right\}d\theta d\varphi \\ =0 \end{array},$$

(A5) $$\begin{array}{l} E[\rho_{i\theta }\rho_{i\varphi }^{*}]=P_{i}\iint E[s_{i}(\theta ,\varphi ,t)(\varphi -\varphi_{i})s_{i}^{*}(\theta ,\varphi ,t)]d\theta d\varphi \\ =P_{i}\iint (\varphi -\varphi_{i})f_{i}(\theta ,\varphi ,t)d\theta d\varphi \\ =\iint \frac{\left(\varphi -\varphi_{i}\right)}{2\pi \sigma_{\theta i}\sigma_{\varphi i}\sqrt{1-\rho^{2}}}\exp\left\{-\frac{1}{2\sigma_{\theta i}\sigma_{\varphi i}(1-\rho^{2})}\left[{\left(\frac{\theta -\theta_{i}}{\sigma_{\theta i}}\right)}^{2}-2\rho \frac{\left(\theta -\theta_{i})(\varphi -\varphi_{i}\right)}{\sigma_{\theta i}\sigma_{\varphi i}}+{\left(\frac{\varphi -\varphi_{i}}{\sigma_{\varphi i}}\right)}^{2}\right]\right\}d\theta d\varphi \\ =0 \end{array}.$$
